# Molecular analysis of Ku redox regulation

**DOI:** 10.1186/1471-2199-10-86

**Published:** 2009-08-28

**Authors:** Sara M Bennett, Tracy M Neher, Andrea Shatilla, John J Turchi

**Affiliations:** 1 Department of Biochemistry & Molecular Biology, Indiana University School of Medicine, Indianapolis, USA; 2 Department of Medicine, Indiana University School of Medicine, Indianapolis, USA

## Abstract

**Background:**

DNA double-strand breaks (DSBs) can occur in response to ionizing radiation (IR), radiomimetic agents and from endogenous DNA-damaging reactive oxygen metabolites. Unrepaired or improperly repaired DSBs are potentially the most lethal form of DNA damage and can result in chromosomal translocations and contribute to the development of cancer. The principal mechanism for the repair of DSBs in humans is non-homologous end-joining (NHEJ). Ku is a key member of the NHEJ pathway and plays an important role in the recognition step when it binds to free DNA termini. Ku then stimulates the assembly and activation of other NHEJ components. DNA binding of Ku is regulated by redox conditions and evidence from our laboratory has demonstrated that Ku undergoes structural changes when oxidized that results in a reduction in DNA binding activity. The C-terminal domain and cysteine 493 of Ku80 were investigated for their contribution to redox regulation of Ku.

**Results:**

We effectively removed the C-terminal domain of Ku80 generating a truncation mutant and co-expressed this variant with wild type Ku70 in an insect cell system to create a Ku70/80ΔC heterodimer. We also generated two single amino acid variants of Cys493, replacing this amino acid with either an alanine (C493A) or a serine (C493S), and over-expressed the variant proteins in SF9 insect cells in complex with wild type Ku70. Neither the truncation nor the amino acid substitutions alters protein expression or stability as determined by SDS-PAGE and Western blot analysis. We show that the C493 mutations do not alter the ability of Ku to bind duplex DNA in vitro under reduced conditions while truncation of the Ku80 C-terminus slightly reduced DNA binding affinity. Diamide oxidation of cysteines was shown to inhibit DNA binding similarly for both the wild-type and all variant proteins. Interestingly, differential DNA binding activity following re-reduction was observed for the Ku70/80ΔC truncation mutant.

**Conclusion:**

Together, these results suggest that the C-terminal domain and C493 of Ku80 play at most a minor role in the redox regulation of Ku, and that other cysteines are likely involved, either alone or in conjunction with these regions of Ku80.

## Background

DNA double strand breaks (DSBs) can be caused by ionizing radiation, reactive oxygen species and other endogenous and exogenous events. If these breaks are not repaired they ultimately result in cell death. Inaccurate repair of these breaks can generate chromosomal translocations, deletions and mutations which can lead to genetic instability and contribute to the development and progression of cancer. There are two main pathways to repair DSBs, homologous recombination (HR) and non-homologous end joining (NHEJ)[1]. HR occurs with minimal loss of genetic material increasing its accuracy and only occurs when a homologous chromosome is present providing extensive regions of sequence homology. NHEJ is error-prone, however it does not require a homologous chromosome or significant regions of homology and is the predominant pathway to repair IR-induced DNA DSBs. NHEJ is initiated upon Ku binding to the DNA termini generated from the DSB. Subsequent binding of the DNA dependent protein kinase catalytic subunit (DNA-PKcs) forms the activated DNA-PK holoenzyme[2]. Active DNA-PK then catalyzes autophosphorylation and phosphorylation of other downstream NHEJ proteins such as Artemis[3], MRE11/RAD50/NBS1 (MRN)[4], and DNA ligase IV/XRCC4 [5].

Ku plays a key role in the NHEJ pathway by binding DNA ends and recruiting other downstream proteins. The crystal structure of Ku revealed a bridge and pillar region comprised of both Ku70 and Ku80 subunits that forms a ring around DNA [6]. These studies revealed the ring shape in the presence and absence of DNA as well as a great deal of structure homology between the two subunits, despite the fact that they share minimal sequence homology[6]. The 3-dimensionial structure of Ku enables the protein to slide or translocate along the length of a DNA molecule[7]. However, it is unclear how Ku dissociates from the DNA upon completion of the NHEJ pathway when the termini are eventually ligated. Additional studies have demonstrated that upon DNA-PKcs binding, Ku translocates inward along the DNA in an ATP independent manner [2] consistent with the sliding model. Studies have shown that Ku binds DNA in a sequence independent fashion by way of several hydrophobic residues that make contact with the major groove of DNA and several basic residues that interact with the phosphate back bone[6,8]. Studies have shown that the Ku70 subunit is proximal to the DSB and Ku80 is distal to the DSB[2].

While much is known about the biochemical activities of Ku, its physiological regulation is less well understood. It has been determined that oxidative stress has a significant effect on the NHEJ pathway. Previous studies have shown that under oxidative conditions there is a marked decrease in DNA-PK activity[9,10]. More specifically, oxidative stress has been shown to impair Ku's ability to bind DNA[11]. Research has indicated a conformational change in Ku under oxidized conditions that leads to a significantly higher K_off _rate [12]. The affect oxidative stress has on Ku is a curious issue when thinking in terms of the crystal structure of Ku. The crystal structure does not reveal any disulfide bonds, however it is lacking several amino acids, particularly a cysteine in the C-terminal region of Ku80.

To further understand how redox conditions influence Ku structure and activity we constructed, purified and characterized several mutants of Ku. These mutations were introduced in key positions of the Ku80 subunit that have been implicated in redox regulation. The results are discussed with respect to the effect of redox on Ku structure and activity.

## Methods

### Materials

Sequencing grade bovine trypsin was purchased from Roche Diagnostics (Indianapolis, IN).

DNA primers and oligonucleotides used in this study were purchased from Integrated DNA Technologies, Inc. (Carolville, IA). *Srf1 *was purchased from Stratagene (La Jolla, CA) and all other restriction enzymes and T4 DNA ligase were purchased from New England Biolabs, Inc. (Ipswich, MA). Mouse monoclonal antibodies Ku (p70) Ab-4 and Ku (p80) Ab-7 were purchased from Neomarker (Fremont, CA).

### Mutant Construction

Ku80ΔC was prepared via PCR cloning using an anti-sense primer inserting a stop codon after amino acid 548 (Table 1). The PCR product was subcloned into pRSET B to introduce an N-terminal six-histidine tag. The tagged construct was subcloned into pBacPak 8 and used to generate a recombinant baculovirus via co-transfection with bacpak6 viral DNA (Clonetech; Mountain View, CA). Following plaque purification and amplification of the Ku80ΔC virus, protein production of the Ku70/80ΔC was achieved by co-infection with wild type Ku 70 virus as previously described [12].

**Table 1:** DNA oligonucleotides

Primer name	Sequence (5'→3')
C493A*	CGATTTCAGAGATTATTTCAGGCTCTGCTGCACAGAGC
C493S*	CGATTTCAGAGATTATTTCAGTCTCTGCTGCACAGAGC
Sense	ATACCGTCCCACCATCGGGC
Antisense	GAATTCCTAAGCAGTCACTTGATCCTTTT
30A	CCCCTATCCTTTCCGCGTCCTTACTTCCCC
30C	GGGGAAGTAAGGACGCGGAAAGGATAGGGG

* The underlined bases indicates the position of the mutation.

Single amino acid substitutions of the human Ku80 protein in the pFastBac1 expression vector were generated using the QuikChange II site-directed mutagenesis kit (Stratagene; La Jolla, Ca). Briefly, for each mutation, the plasmid was PCR-amplified using two complementary oligonucleotide primers containing the desired mutation (Table 1). The PCR products were treated with Dpn1 to degrade the methylated parental DNA template. The DNA was then amplified and recombinant baculovirus was generated in the Bac-to-Bac Baculovirus expression system (Invitrogen; Carlsbad, CA). The pFastBac1 expression vector containing the mutated Ku80 gene, was transformed into *E. coli *strain DH10Bac that contains a baculovirus shuttle vector, bacmid. Transformants were selected and high molecular weight recombinant bacmid DNA was extracted and used for transfection of SF9 cells using FuGENE 6. The clarified transfection supernatant, containing the recombinant baculovirus, was plaque purified as needed and recombinant virus was amplified. Protein expression was accomplished via co-infection with Ku70 virus as previously described [12,13].

### Protein Expression and Purification

Human Ku was purified from Sf9 cells infected with recombinant baculovirus. Cells were infected for 48 hours, and cell-free extracts were prepared. Wild type, Ku70/80ΔC and Ku70/C493 mutants were purified by sequential Ni-NTA and Q-Sepharose column chromatography as previously described [12,14]. Fractions containing Ku were identified based on SDS-PAGE and visualized by Coomassie Blue staining. Peak fractions were pooled, dialyzed overnight and stored at -80°C

### SDS-PAGE and Western Blot

Proteins were separated via SDS-PAGE. Gels were either stained with Coomassie Blue or transferred to PVDF membrane for Western blot analysis. Membranes were blocked with 2% non-fat dry milk in TBS-Tween and probed with the primary antibodies indicated in the figure legends. Bound antibodies were detected with a horse radish peroxidase (HRP) conjugated goat-anti-mouse IgG and visualized via chemiluminescence detection capturing images via a Fuji LAS-3000 CCD system.

### EMSA

Electrophoretic Mobility Shift Assays (EMSAs) were performed as previously described [13,15]. Briefly, reactions were performed in a volume of 20 μl containing 50 mM Tris-Cl pH7.8, 10 mM MgCl_2 _and 50 mM NaCl. Oxidized conditions were achieved by incubating Ku for 15 min on ice in 2 mM Diamide. Re-reduced conditions were achieved by incubating oxidized Ku with 5 mM DTT for 15 min on ice. The protein preparations were then assessed for DNA binding activity in an EMSA containing 500 fmol of ^32^P-labeled 30-bp double strand DNA as previously described using oligonucleotides 30A and 30C (Table 1)[16,17]. Reaction products were then separated by electrophoresis on a 6% native polyacrylamide gel. The gels were then dried and exposed to a PhosphorImager screen (Amersham Biosciences; Piscataway, NJ) and quantified using ImageQuant software. Quantification of the data is presented as the averages and standard deviations of at least three independent measurements. Binding data were fit to the equation 1 describing a sigmoidal curve and K_D _values calculated from the fit of the curve using SigmaPlot software (Systat Software inc. Chicago, IL).

(1)

### Fluorescence Polarization

Fluorescence polarization experiments were performed in 0.5 ml buffer A (50 mM Tris -HCl, pH 7.8, 10 mM MgCl_2_s, 50 mM NaCl, 1 mM DTT) using a Cary Eclipse Fluorescence Spectrophotometer (Varian; Palo Alto, CA). Oxidized conditions and re -reduced Ku preparations were generated as described above. The protein preparations were then assessed for DNA binding using a 5'-Fluorscein -labeled 30-bp double-strand DNA as previously described (Table 1)[16,17]. Fluorescence excitation and emission was measured at 495 and 515 nm, respectively. Results are presented as the averages and standard deviations of at least three independent measurements. K_D _values were obtained from fitting the data to sigmoidal curves described above. The data obtained from binding under oxidized conditions were not suitable for K_D _determination.

### Limited Proteolysis

Limited tryptic proteolysis was performed according to established procedures with the following modification [13]. The Ku preparations were analyzed for potential structural changes under control, reduced, conditions and following oxidation and re-reduction. Ku protein preparations (4 μg), prepared as stated above, were subjected to limited proteolysis by the addition of 200 ng of sequencing grade bovine trypsin. Reactions were performed in buffer A and incubated at 37°C for10 minutes. Reactions were terminated by the addition of SDS loading dye and samples were separated by SDS-PAGE. Products were visualized via Coomassie Blue staining and images were captured using Image Reader LAS-3000 and visualized and quantified using MultiGuage V3.0.

## Results

### Identification and mutation of potential redox regulated sites in Ku

**Figure 1:**
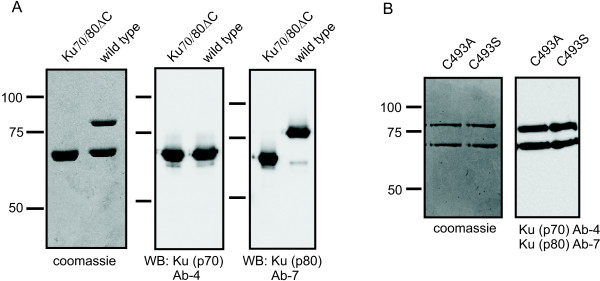
**Purity and stoichiometry of Ku heterodimer complexes**. A) The Ku70/80ΔC and wild type Ku protein preparations were subject to SDS-PAGE and proteins detected by staining with Coomassie Blue, transferred to PVDF and detected with Ku (p70) Ab-4 or Ku (p80) Ab-7 antibodies as indicated in the figure. B) The C493A and C493S point mutants were also analyzed by SDS-PAGE and western blot analysis.

Our previous analyses indicated that specific regions or domains of Ku may be involved in redox regulation of Ku DNA binding activity. We have reported alterations in Ku structure as a function of redox conditions and identified C493 of Ku80 as an accessible cysteine position in a region where a modification of cysteine chemistry could potentially influence DNA binding. Chemical reactivity probes also identified the C-terminal domain of Ku which contains C638 as an area of interest that has the potential to influence the DNA binding activity of Ku [13]. Therefore we prepared two mutants of C493 replacing this amino acid with an alanine or a serine. The serine substitution retains the potential to serve as a hydrogen bond donor, while the alanine does not. Both alanine and serine are not sensitive to redox conditions and thus provide an assessment of the role of C493, if any, in Ku redox regulation. To assess the role of the C-terminus of Ku80 and C638, we prepared a truncation mutant of Ku80 via PCR primers designed to introduce a stop codon after amino acid 548. Each construct was used to generate a recombinant baculovirus and co-expressed with wild type his -tagged Ku70. The recombinant proteins were purified via Ni-agarose column chromatography followed by fractionation on a Macro-prep Q anion exchange matrix. SDS-PAGE analysis of the purified proteins is presented in Figure 1. The results demonstrate a 1:1 stoichiometry for each preparation which was confirmed by western blot analysis. This was especially important for the Ku70/80ΔC construct where the truncated Ku80 migrates very closely to the [His]_6_-tagged Ku70 as judged on a Coomassie Blue stained gel. The reactivity of this protein with an antibody to the Ku80 N-terminus confirmed co-expression and 1:1 stoichiometry (Figure 1A). Analysis of the C493 mutants also demonstrated 1:1 stoichiometry and no alterations in protein expression or degradation as a result of this change (Figure 1B).

### DNA binding of Ku is independent of the Ku 80 CTD and C493

**Figure 2:**
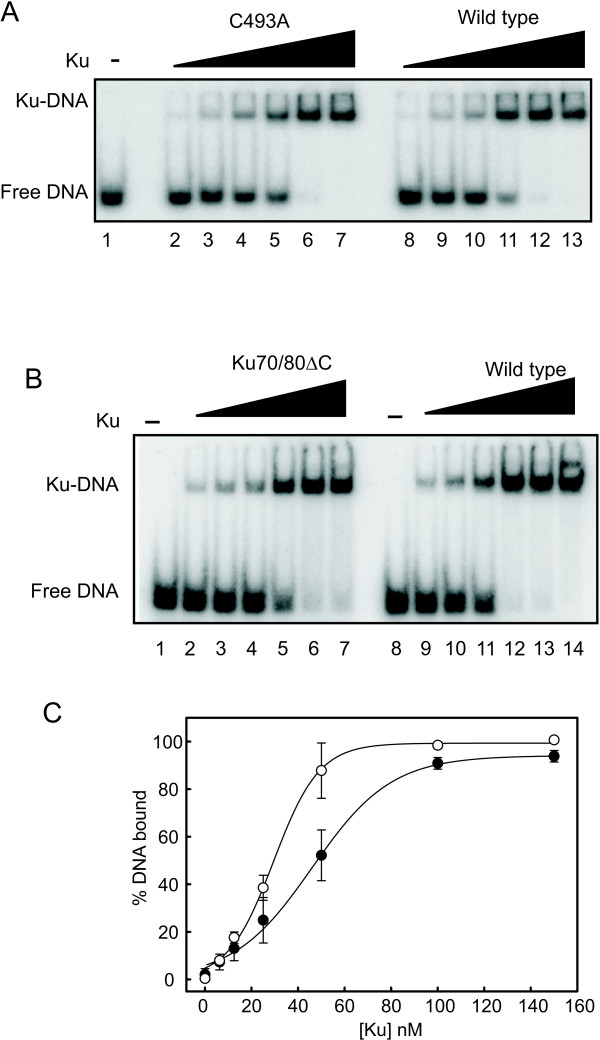
**DNA binding of Ku mutants under reduced conditions as assessed by EMSA**. (A) C493A point mutant and wild type Ku were assessed for binding to a duplex 30-bp DNA substrate as described in "Methods". Reaction products were separated via native electrophoresis and visualized via PhosphorImager. (B) Ku70/80ΔC and wild type Ku (0-150 nM) were subject to the same analysis. (C) Quantification of the results in Panel B was performed via PhosphoImager analysis. Results are presented as the average and standard deviation of triplicate determinations and binding curves were fit. Filled circles represent wild type Ku, open circles represent Ku70/80ΔC.

DNA binding of the purified Ku variants was assessed in EMS As using a 30-bp duplex DNA under reduced conditions, which we have shown allow for maximal DNA binding activity of Ku [7,15,18]. Analysis of the C493A variant (Figure 2A) demonstrates near wild type binding to duplex DNA, indicating that the mutation to an alanine does not influence the interaction with DNA under reduced conditions. Similar results were observed with the C493S mutant with binding being similar to wild type (data not shown). The results presented in Figure 2B demonstrate that the Ku70/80ΔC variant lacking the C-terminal domain is capable of binding DNA with only as light reduction in binding affinity compared to wild type Ku (Table 2). Quantification of the data is presented in Figure 2C. While this C-terminal domain has been demonstrated to be involved in activation of DNA-PKcs[19], these results demonstrate that DNA binding is only moderately affected by removal of this domain, a result consistent with the crystal structure of the Ku heterodimer bound to DNA [6]. These data support the contention that no dramatic alteration in DNA binding activity is manifested by the introduced changes in the protein's primary structure.

### Redox effects on DNA Binding

**Figure 3:**
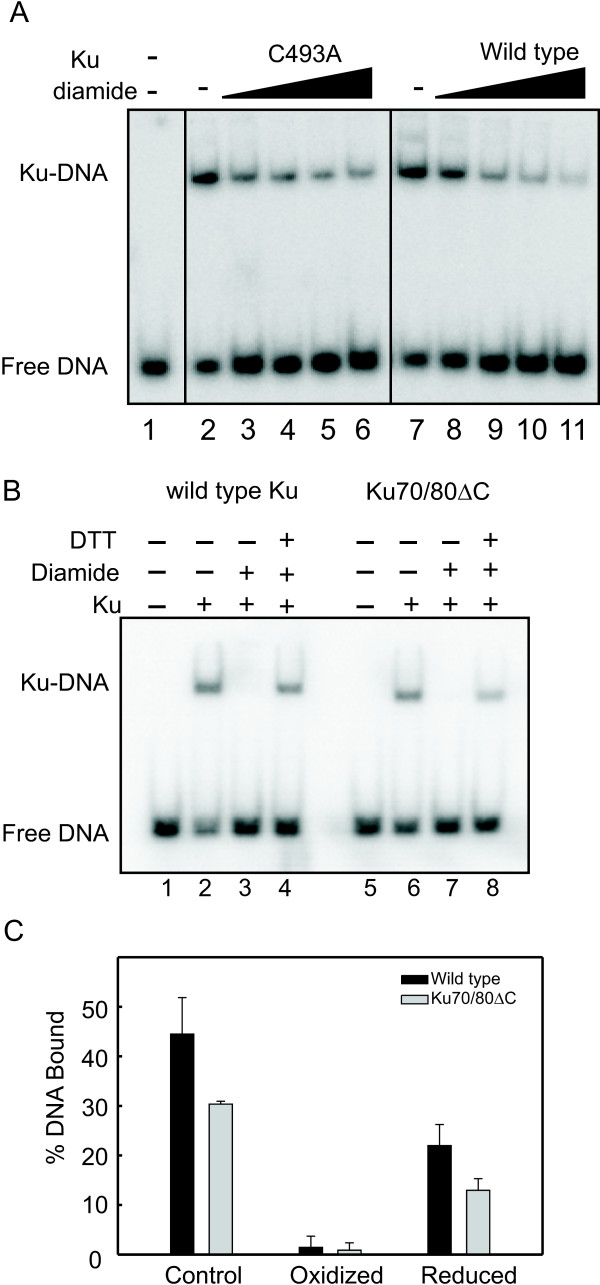
**The effect of oxidation on DNA binding of Ku mutants**. (A) C493A point mutant and wild type Ku was exposed to increasing concentrations of diamide (0-2 mM) and then DNA binding activity assessed. (B) Wild type and Ku70/80ΔC (950 nM) were incubated with 2 mM diamide and DNA binding assessed, For re-reduced conditions, diamide incubation was followed by incubation with 5 mM DTT. DNA binding was then assessed as described in the legend for Figure 2. (C) Quantification of the results in Panel B was performed via PhosphorImager analysis. Results are presented as the average and standard deviation of triplicate determinations.

To assess the effect of redox on Ku binding we used the cysteine specific oxidant, diamide, to oxidize Ku and then assessed binding in are action performed in the absence of added DTT. Previously we have shown that under these conditions Ku exhibits a reversible oxidation event that impairs DNA binding [12]. The results presented in Figure 3 demonstrate that wild type Ku displays a decrease in DNA binding activity as a function of diamide concentration, consistent with our previous results[12].

**Figure 4:**
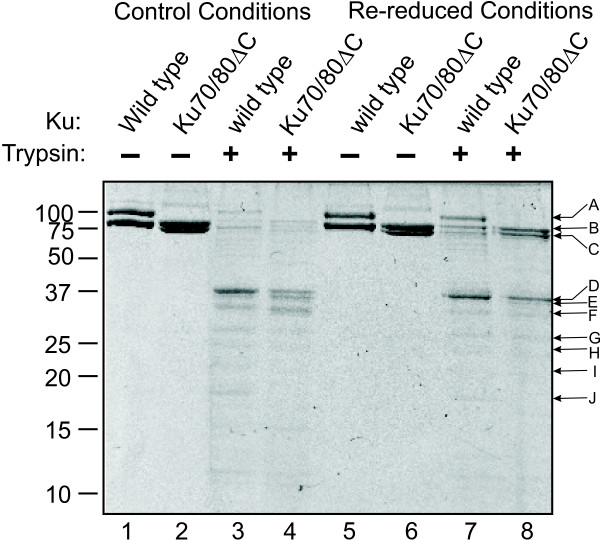
**Effect of oxidation on Ku mutant structure**. Following oxidation and re-reduction as described in the legend to Figure 3, wild type Ku (4 μg) and Ku70/80ΔC (4 μg) were subjected to limited tryptic cleavage. Diamide was added after the addition of DTT to the control reactions while reduced conditions were first oxidized with diamide then re-reduced with DTT. Reactions were terminated by the addition of SDS sample buffer and products separated by SDS-PAGE and visualized via staining with Coomassie Blue.

**Figure 5:**
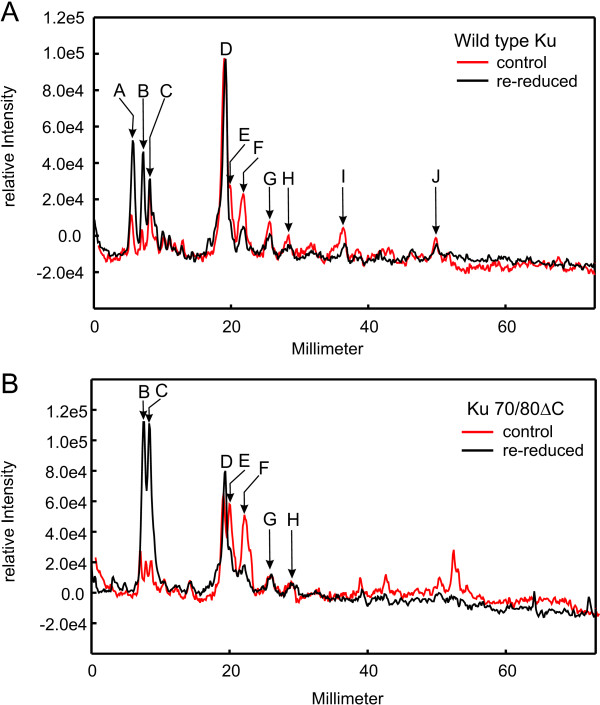
**Quantification of Limited Proteolysis**. Band intensity was assessed via digitization of the gel image using Fuji Multigauge software. The intensity was normalized and plotted versus distance for the indicated lanes. (A) Wild type Ku digested with trypsin under control and re-reduced conditions, (B) Ku70/80ΔC digested with trypsin under control and re-reduced conditions.

Interestingly, the C493A mutant also exhibited a decrease in binding as a function of redox conditions (Figure 3A). These results suggest that the redox-dependent inhibition of Ku DNA binding activity is independent of C493. A similar line of experimentation was performed with the truncation mutant (Figure 3B) and again Ku binding was reduced in both protein preparations as a function of diamide. We also reversed the conditions to re-reduce the protein by incubation with additional DTT. The result of this treatment was the restoration of DNA binding activity for the wild type protein and Ku 70/80ΔC truncation variant. Interestingly, full DNA binding activity was not observed for either protein preparation, thus the potential for a persistent redox-dependent structural change exists in Ku (Figure 3C). To determine if in fact a persistent structural alteration in Ku exists following diamide oxidation and re-reduction, we performed a limited tryptic digest of the Ku that was treated with DTT before diamide addition at a ratio that does not result in Ku oxidization (control) and Ku that was first treated with diamide to oxidize the proteins was then re-reduced with DTT. The final concentration of all components was identical prior to the tryptic cleavage reactions. The results presented in Figure 4 demonstrate that there is somewhat more tryptic cleavage under the control conditions compared to the re-reduced Ku and this is apparent in both the wild-type and Ku70/80ΔC mutant. This is also apparent in the prominence of the lower molecular mass peptides (<37 kDa) under control conditions and the prominence of the full length proteins in the re-reduced conditions. This is somewhat surprising in that the better DNA binding conditions (control) result in greater tryptic susceptibility. Quantification of the data, which is presented in Figure 5, bears out the interpretation that re-reduced Ku is slightly less susceptible to tryptic cleavage. A difference in peptide product distribution was observed between the wild type and Ku70/80ΔC under both conditions that could not be attributed to the truncation. This suggests that the folding or conformation of the Ku70/80ΔC protein differs from that of wild type Ku. Interestingly this minor difference may account for the difference in DNA binding affinity observed in the EMSA DNA binding analysis of the Ku70/80ΔC.

**Figure 6:**
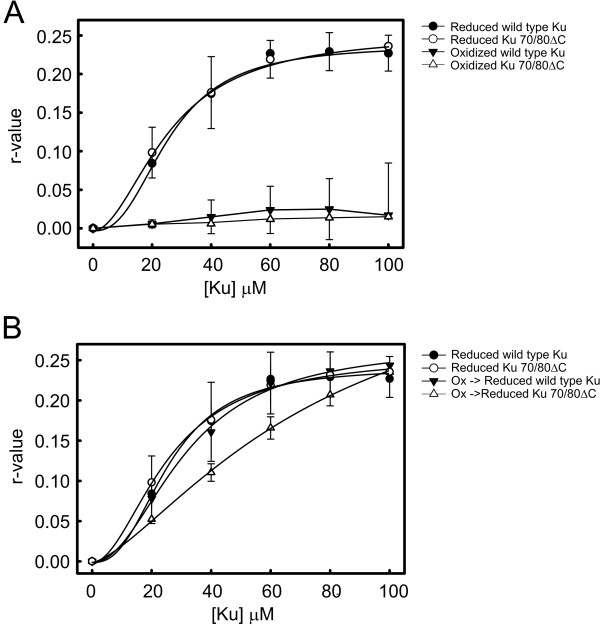
**Anisotropy Assay of Wild Type and Ku70/80ΔC under (A) Control verses Oxidized Conditions and (B) Control verses Re-reduced**. Anisotropy assay were performed in triplicate as described in methods and materials. Data presented represent averages and standard deviations of these determinations. Ku preparations used in each series are designated in each figure.

While the ability of diamide to reversibly oxidize the Ku variants suggests that C493 and C638 are not involved in redox regulation, we have previously demonstrated that solution based true equilibrium binding assays can often allow differences in activity to be determined that are not observed in EMS As. Therefore we assessed binding of wild type and Ku variants to a duplex DNA substrate using a fluorescence polarization assay. A 30-bp duplex with a single fluoroscein label on one 5' terminus was used and Ku protein preparations were titrated into the binding reactions. Wild type and Ku mutants under reduced conditions displayed identical binding and, upon oxidation with diamide, minimal DNA binding was detected (Figure 6A, Table 2 and data not shown for C493 mutants). The oxidized Ku proteins where then re-reduced by the addition of excess DTT and titrated into binding reactions. In this analysis, wild type Ku regained complete DNA binding activity while the Ku70/80ΔC again regained significant DNA binding activity, though not complete. This data is consistent with the alteration in trypsin sensitivity observed following re-reduction after oxidation such that after re-reduction both wild type and Ku70/80ΔC were less sensitive to trypsin digestion and suggest an alteration in structure that impacts DNA binding activity. Overall, these analyses provide insight into how Ku structural features impact redox regulation. Amore complete understanding of this complex interplay is likely to require additional genetic and structural analyses as well as an investigation of cellular redox regulators that may perform this function *in vivo*.

**Table 2:** K_D _Values as determined by EMSA and anisotropy

	Conditions	K_D _(μM)	B_Max_	R value
*EMSA*				
Wild type Ku	Control	29.7 ± 2.5	--	0.99
Ku70/80ΔC	Control	51.0 ± 7.7	--	0.99

*Anisotropy*				
Wild type Ku	Control	25.8 ± 2.3	0.24	0.995
	Oxidized	--	--	--
	Re-reduced	31.4 ± 3.0	0.20	0.98
Ku70/80ΔC	Control	25.7 ± 1.5	0.255	0.999
	Oxidized	--	--	--
	Re-reduced	<50	--	--

## Discussion

It has been previously shown that the Ku-DNA interaction is favored under reduced conditions[11,12,20]. Physiologically, Ku, DNA-PK and NHEJ have all been demonstrated to be influenced by cellular redox conditions[9,10,21]. Typically, redox dependent alterations in enzymatic or binding activity can be attributed to the formation and breaking of disulfide bonds. Interestingly, X-ray structural analysis of Ku does not reveal the presence of any disulfide bonds [6]. Therefore, the oxidation effects observed *in vitro *are likely to be a result of the formation of cysteine sulfenic acids. This reversible modification is consistent with the near complete rescuing of DNA binding activity upon re-reduction (Figure 3 and ref [12]). Cellular modifications as a function of redox however, may be manifested by different modifications and interactions.

Specifically, glutathionine conjugations to Ku could be responsible for the decreased binding activity observed in cells exposed to ROS via treatment with glucose and glucose oxidase[21]. Also, protein-protein disulfide bonds between exposed cysteine residues could account for the reduction in binding. This, however, is unlikely to account for all the reduction as the Ku protein level was reduced and not observed as highly cross linked [21]. Preliminary data demonstrate that at least 7 cysteine residues are readily accessible, one being located in the C-terminal domain, and thus glutathionine conjugates or modification to sulfenic acid could account for the reduction in DNA binding activity. While the results presented demonstrate that C493 and C638 are not likely to be the major determinants of redox regulation, the inability to completely re-reduce Ku70/80ΔC suggested that C638 may play some role in redox dependent changes in Ku structure.

The DNA binding assays performed on the C493 mutants and Ku70/80ΔC under oxidized conditions revealed a near complete loss of DNA binding activity. If C493 and 638 were responsible for the redox regulation, no reduction in DNA binding activity would have been observed. Demonstration of the loss of binding in two independent assays provides strong evidence that upon oxidation, the protein structure is altered such that it cannot support DNA binding. When the proteins were re -reduced, the DNA binding ability was largely recovered, again consistent with a modification or alteration in structure being reversible. Also, in that the wild type Ku and Ku 70/80ΔC behaved similarly in their recovery suggest that the Ku80CTD does not protect any cysteine residues from the redox conditions that affect Ku's ability to bind DNA. While we observed some discrepancies between the wild type Ku and Ku70/80ΔC anisotropy data and EMSA data, this could be explained in the nature of these assays. The anisotropy assay is more representative of a true steady state equilibrium binding assay, where as the EMSA is a stopped assay with post -binding separation that can influence the detection of the bound species. This is apparent from the fits of the binding curves for each assay where the EMSA quantification yields a clear sigmoidal binding curve as we have previously demonstrated for Ku binding short DNA duplexes [18]. The data obtained from fluorescence anisotropy fits better to a hyperbolic binding curve than the EMSA data, consistent with a true equilibrium binding reaction (data not shown). Despite these differences, the results of the redox effects between the wild type and variant proteins are very consistent.

## Conclusion

In conclusion we observed that under oxidized conditions Ku, independent of the mutations, binds DNA with a significantly lower affinity than under reduced conditions. We also determined the C493 does not play a role in Ku-DNA interaction nor does it have a role in the affect of redox on the Ku heterodimer. Analysis of the Ku70/80ΔC revealed slight differences in activity leading us to believe that this truncation has a modest effect on Ku binding DNA and potentially a similar effect on Ku structure and activity in response to redox conditions.

## Authors' contributions

SMB prepared and performed EMSA analysis of the Ku truncation mutant and prepared the first draft of the manuscript. TMN performed all fluorescence polarization and limited proteolysis experiments and participated in the writing and editing of this manuscript. AS prepared and performed the analysis of the Ku point mutants and edited the manuscript. JJT supervised all experimentation, edited and prepared the final draft of the manuscript. All authors have read and approved the final manuscript
